# The genome sequence of the two-spot ladybird, *Adalia bipunctata *(Linnaeus, 1758)

**DOI:** 10.12688/wellcomeopenres.18610.1

**Published:** 2022-11-30

**Authors:** Wellcome Sanger Institute Tree of Life programme, Wellcome Sanger Institute Scientific Operations: DNA Pipelines collective, Tree of Life Core Informatics collective, Zoe Goate, Darwin Tree of Life Consortium

**Affiliations:** 1 Wellcome Sanger Institute, Hinxton, UK

**Keywords:** Adalia bipunctata, two-spot ladybird, chromosomal, Coleoptera

## Abstract

We present a genome assembly from an individual male *Adalia bipunctata* (the two-spot ladybird; Arthropoda; Insecta; Coleoptera; Coccinellidae). The genome sequence is 475 megabases in span. Most of the assembly (94.87%) is scaffolded into 11 chromosomal pseudomolecules, with the X and Y sex chromosomes assembled. The complete mitochondrial genome was also assembled and is 21.2 kilobases in length. Gene annotation of this assembly in Ensembl identified 13,611 protein coding genes.

## Species taxonomy

Eukaryota; Metazoa; Ecdysozoa; Arthropoda; Hexapoda; Insecta; Pterygota; Neoptera; Endopterygota; Coleoptera; Polyphaga; Cucujiformia; Coccinellidae; Coccinellinae; Coccinellini; Adalia; *Adalia bipunctata* (Linnaeus, 1758) (NCBI:txid7084).

## Background

**Figure 1.**
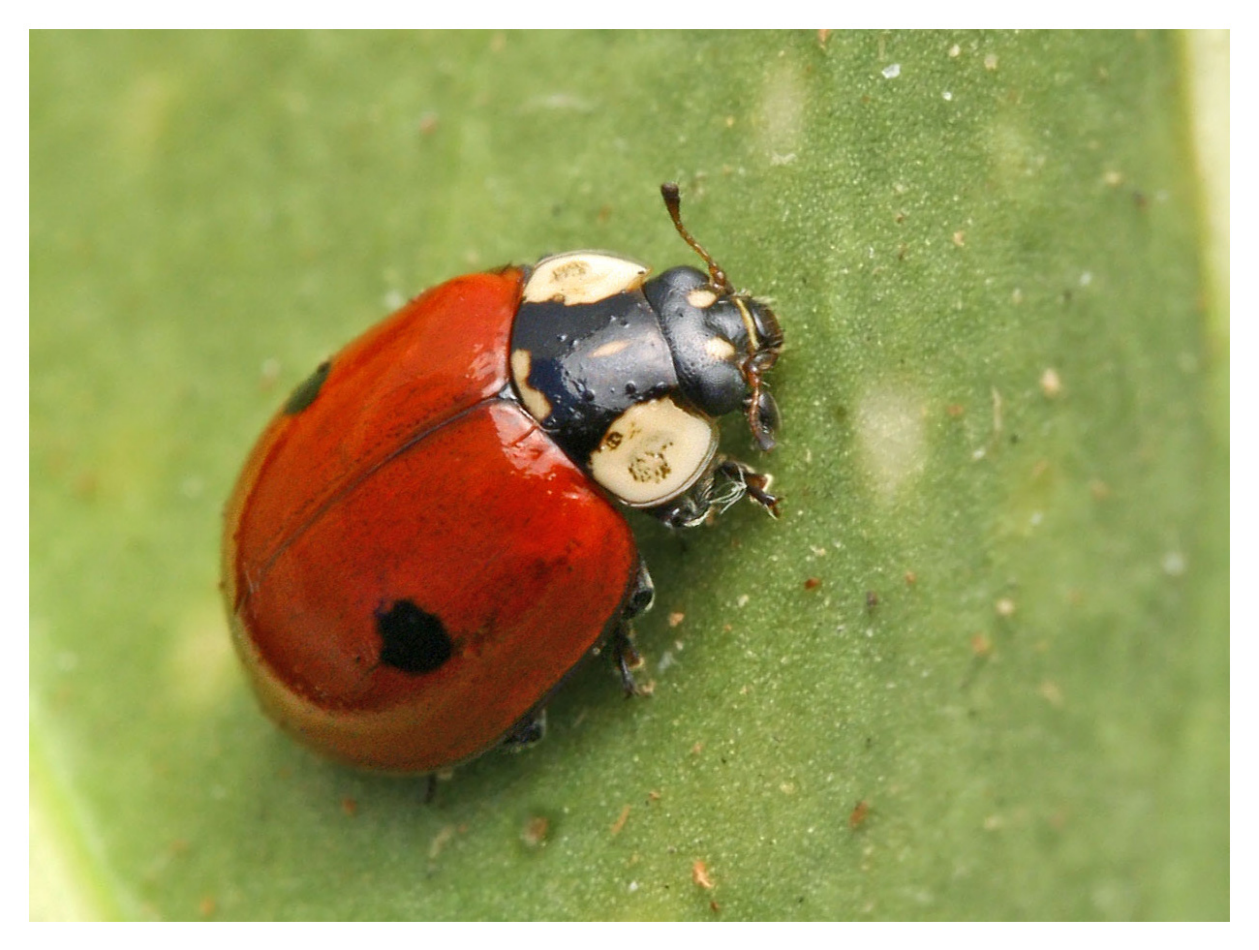
An *Adalia bipunctata* image (Photograph from www.entomart.be CC-BY).

The two-spot ladybird, *Adalia bipunctata* (Linnaeus, 1758) is a Holarctic species native to Europe, Central Asia and North America. *A. bipunctata* was once the second most common ladybird in the US, but the invasion of the predatory Asian species, the harlequin ladybird, *Harmonia axyridis* has seen a rapid decline in the two-spot population over the last decade ( Kenis *et al.*, 2020). This widespread species occupies a variety of habitats, from deciduous or coniferous woodlands to orchards and crops. In temperate regions, adults appear in March and are known to overwinter in large groups along with other common species in among loose bark, leaf-litter and outhouses. Both adult and larval forms of *A. bipunctata* are voracious aphidophagous hunters, making them suitable biocontrol agents against aphids in agricultural systems ( Riddick, 2017). Two-spots exhibit complex polymorphism with typical morphs conspicuously marked with vivid red elytra and a large black spot in the middle of each ( Figure 1), whilst melanic morphs display a black elytra with red spots ( Rutkowski *et al.*, 2019).

The two-spot ladybird is a classic model for population genetics studies, and a complete genome assembly of *A. bipunctata* may help to characterise the genetic diversity underpinning phenotypic polymorphisms among populations across different environments ( Gautier *et al.*, 2018).

We present a complete genome assembly for *A. bipunctata* as part of the Darwin Tree of Life project, which aims to sequence the genomes of 70,000 species of eukaryotic organisms in Britain and Ireland.

## Genome sequence report

**Figure 2.**
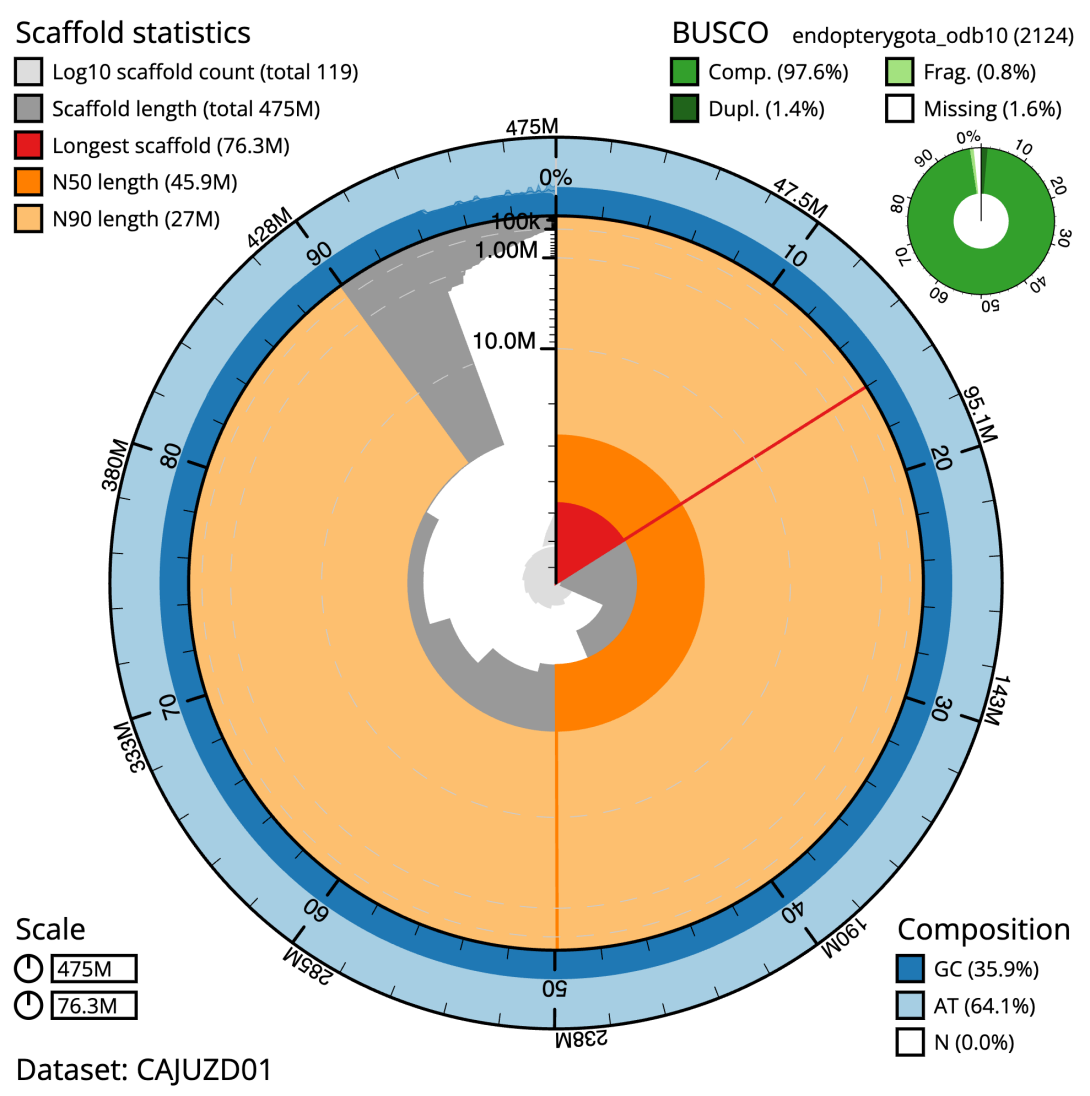
Genome assembly of *A. bipunctata*, icAdaBipu1.1: metrics. The BlobToolKit Snailplot shows N50 metrics and BUSCO gene completeness. The main plot is divided into 1,000 size-ordered bins around the circumference with each bin representing 0.1% of the 475,288,177 bp assembly. The distribution of chromosome lengths is shown in dark grey with the plot radius scaled to the longest chromosome present in the assembly (76,338,783 bp, shown in red). Orange and pale-orange arcs show the N50 and N90 chromosome lengths (45,869,983 and 27,047,566 bp), respectively. The pale grey spiral shows the cumulative chromosome count on a log scale with white scale lines showing successive orders of magnitude. The blue and pale-blue area around the outside of the plot shows the distribution of GC, AT and N percentages in the same bins as the inner plot. A summary of complete, fragmented, duplicated and missing BUSCO genes in the endopterygota_odb10 set is shown in the top right. An interactive version of this figure is available at https://blobtoolkit.genomehubs.org/view/icAdaBipu1.1/dataset/CAJUZD01/snail.

The genome was sequenced from an individual male *A. bipunctata* (icAdaBipu1) purchased live from Dragonfli, Essex, UK. A total of 48-fold coverage in Pacific Biosciences single-molecule HiFi long reads and 80-fold coverage in 10X Genomics read clouds were generated. Primary assembly contigs were scaffolded with chromosome conformation Hi-C data. Manual assembly curation corrected 72 missing/misjoins and removed 17 haplotypic duplications, reducing the assembly size by 2.45% and the scaffold number by 34.08%, and increasing the scaffold N50 by 105.95%. The final assembly has a total length of 475 Mb in 118 sequence scaffolds with a scaffold N50 of 45.9 Mb ( Table 1). Most of the assembly sequence (94.87%) was assigned to 11 chromosomal-level scaffolds, representing 9 autosomes (numbered by sequence length), and the X and Y sex chromosomes ( Figure 2– Figure 5; Table 2).

**Table 1.** Genome data for *A. bipunctata*, icAdaBipu1.

*Project accession data*	
Assembly identifier	icAdaBipu1.1
Species	*Adalia bipunctata*
Specimen	icAdaBipu1
NCBI taxonomy ID	7084
BioProject	PRJEB45109
BioSample ID	SAMEA9089055
Isolate information	Male, whole organism
*Raw data accessions*	
PacificBiosciences SEQUEL II	ERR7015066
10X Genomics Illumina	ERR6842405–ERR6842408
Hi-C Illumina	ERR9866423–ERR9866427
*Genome assembly*	
Assembly accession	GCA_910592335.1
*Accession of alternate haplotype*	GCA_910591895.1
Span (Mb)	475
Number of contigs	212
Contig N50 length (Mb)	18.3
Number of scaffolds	118
Scaffold N50 length (Mb)	45.9
Longest scaffold (Mb)	76.3
BUSCO * genome score	C:97.6%[S:96.1%,D:1.4%], F:0.8%,M:1.6%,n:2124
*Genome annotation*	
Number of protein-coding genes	13,611

*BUSCO scores based on the endopterygota_odb10 BUSCO set using v5.2.2. C = complete [S = single copy, D = duplicated], F = fragmented, M = missing, n = number of orthologues in comparison. A full set of BUSCO scores is available at https://blobtoolkit.genomehubs.org/view/icAdaBipu1.1/dataset/CAJUZD01/busco.

**Figure 3.**
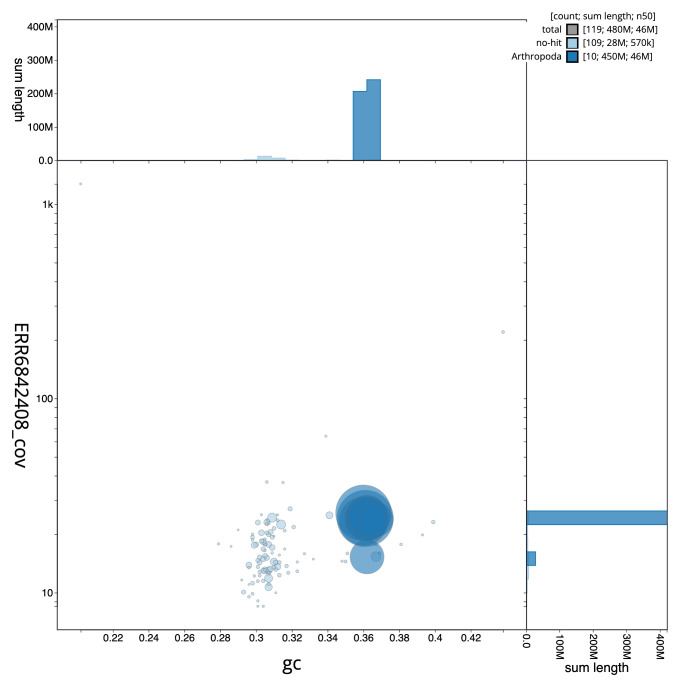
Genome assembly of *A. bipunctata*, icAdaBipu1.1: GC coverage. BlobToolKit GC-coverage plot. Scaffolds are coloured by phylum. Circles are sized in proportion to scaffold length. Histograms show the distribution of scaffold length sum along each axis. An interactive version of this figure is available at https://blobtoolkit.genomehubs.org/view/icAdaBipu1.1/dataset/CAJUZD01/blob.

**Figure 4.**
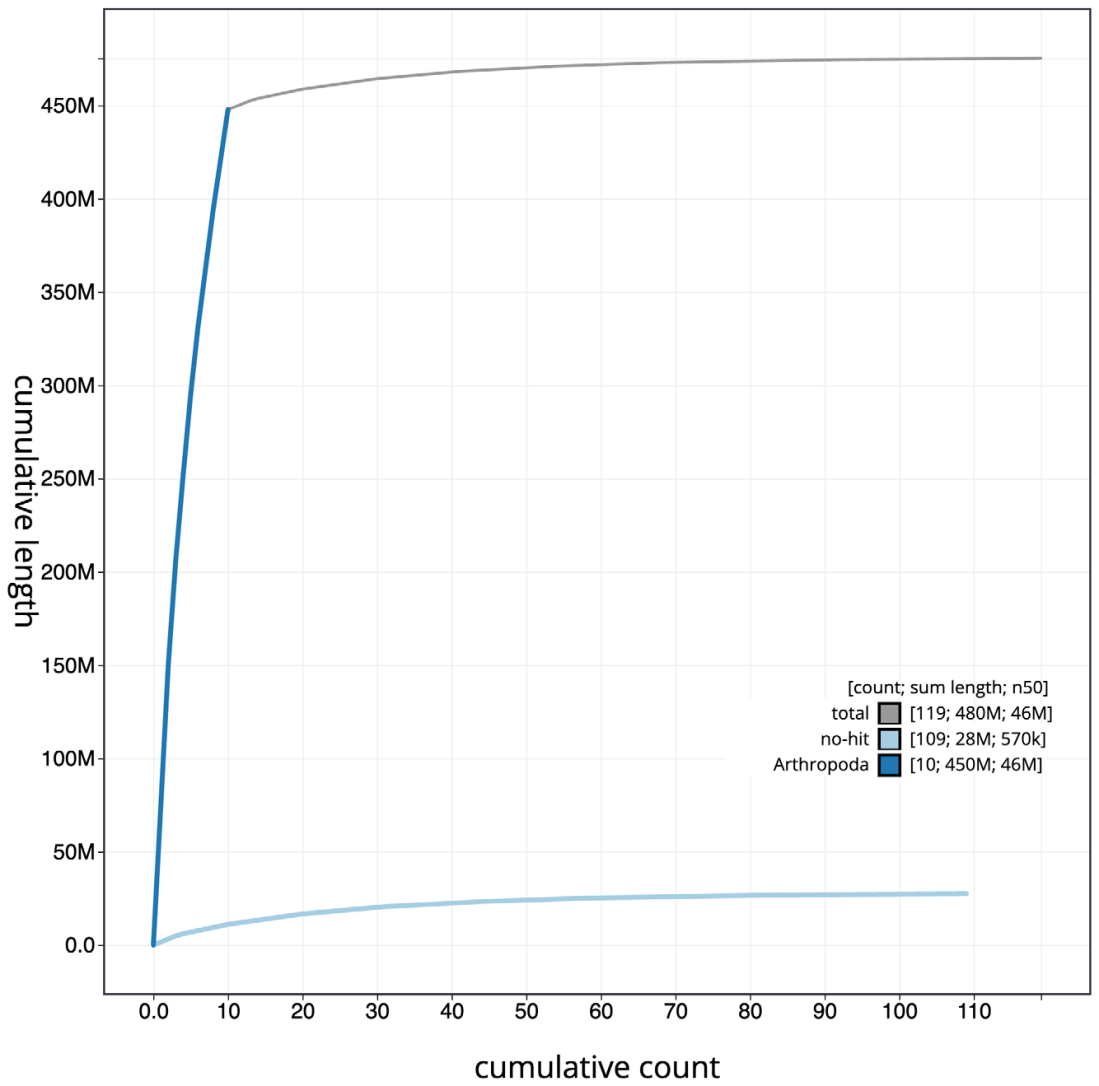
Genome assembly of *A. bipunctata*, icAdaBipu1.1: cumulative sequence. BlobToolKit cumulative sequence plot. The grey line shows cumulative length for all scaffolds. Coloured lines show cumulative lengths of scaffolds assigned to each phylum using the buscogenes taxrule. An interactive version of this figure is available at https://blobtoolkit.genomehubs.org/view/icAdaBipu1.1/dataset/CAJUZD01/cumulative.

**Figure 5.**
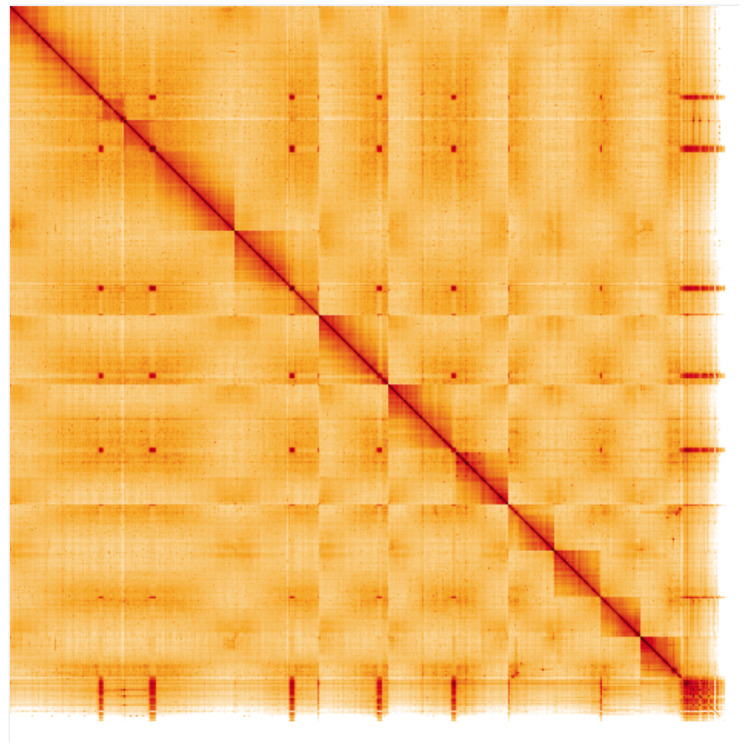
Genome assembly of *A. bipunctata,* icAdaBipu1.1: Hi-C contact map. Hi-C contact map of the icAdaBipu1.1 assembly, visualised in HiGlass. Chromosomes are arranged in size order from left to right and top to bottom. An interactive version of this image can be viewed at https://genome-note-higlass.tol.sanger.ac.uk/l/?d=HVP-dSN3RVK70oemlLsXTA.

**Table 2.** Chromosomal pseudomolecules in the genome assembly of *A. bipunctata*, icAdaBipu1.1.

INSDC accession	Chromosome	Size (Mb)	GC%
OU342948.1	1	76.34	36.1
OU342949.1	2	74.05	36.0
OU342950.1	3	55.99	36.0
OU342951.1	4	45.87	36.2
OU342952.1	5	43.01	36.2
OU342953.1	6	36.65	36.2
OU342954.1	7	30.9	36.3
OU342955.1	8	30.76	36.2
OU342956.1	9	27.12	36.7
OU342957.1	X	27.05	36.2
OU342958.1	Y	1.75	36.7
OU342959.1	MT	0.02	20.3
-	unplaced	25.78	31.2

The assembly has a BUSCO v5.2.2 ( Manni *et al.*, 2021) completeness of 97.6% (single 96.2%, duplicated 1.4%) using the endopterygota_odb10 reference set ( *n* = 2,124).

While not fully phased, the assembly deposited is of one haplotype. Contigs corresponding to the second haplotype have also been deposited.

## Genome annotation report

The *A. bipunctata* genome was annotated using the Ensembl rapid annotation pipeline ( Table 1; https://rapid.ensembl.org/Adalia_bipunctata_GCA_910592335.1/Info/Index). The resulting annotation includes 26,646 gene transcripts from 13,611 protein-coding genes and 3,277 non-coding genes.

## Methods

### Sample acquisition and DNA extraction

One male *A. bipunctata* specimen (icAdaBipu1), purchased live from Dragonfli, Essex, UK, was used for this genome assembly. The specimen was preserved on dry ice. DNA was extracted at the Tree of Life laboratory, Wellcome Sanger Institute. The icAdaBipu1 sample was weighed and dissected on dry ice with tissue set aside for Hi-C sequencing. Whole tissue was disrupted using a Nippi Powermasher fitted with a BioMasher pestle. Fragment size analysis of 0.01–0.5 ng of DNA was then performed using an Agilent FemtoPulse. High molecular weight (HMW) DNA was extracted using the Qiagen MagAttract HMW DNA extraction kit. Low molecular weight DNA was removed from a 200 ng aliquot of extracted DNA using 0.8X AMpure XP purification kit prior to 10X Chromium sequencing; a minimum of 50 ng DNA was submitted for 10X sequencing. HMW DNA was sheared into an average fragment size of 12–20 kb in a Megaruptor 3 system with speed setting 30. Sheared DNA was purified by solid-phase reversible immobilisation using AMPure PB beads with a 1.8X ratio of beads to sample to remove the shorter fragments and concentrate the DNA sample. The concentration of the sheared and purified DNA was assessed using a Nanodrop spectrophotometer and Qubit Fluorometer and Qubit dsDNA High Sensitivity Assay kit. Fragment size distribution was evaluated by running the sample on the FemtoPulse system. 

### Sequencing

Pacific Biosciences HiFi circular consensus and 10X Genomics Chromium read cloud sequencing libraries were constructed according to the manufacturers’ instructions. Sequencing was performed by the Scientific Operations core at the Wellcome Sanger Institute on Pacific Biosciences SEQUEL II (HiFi) and Illumina HiSeq X (10X) instruments. Hi-C data were generated in the Tree of Life laboratory from remaining tissue of icAdaBipu1 using the Arima v2 kit and sequenced on a HiSeq X instrument.

### Genome assembly

Assembly of PacBio reads was carried out with Hifiasm ( Cheng *et al.*, 2021) and haplotypic duplication was identified and removed with purge_dups ( Guan *et al.*, 2020). One round of short-read polishing was performed by aligning 10X Genomics read data to the assembly with longranger align, calling variants with freebayes ( Garrison & Marth, 2012). The assembly was then scaffolded with Hi-C data ( Rao *et al.*, 2014) using SALSA2 ( Ghurye *et al.*, 2019). The assembly was checked for contamination as described previously ( Howe *et al.*, 2021). Manual curation was performed using and gEVAL ( Chow *et al.*, 2016), HiGlass ( Kerpedjiev *et al.*, 2018) and Pretext ( Harry, 2022). The mitochondrial genome was assembled using MitoHiFi ( Uliano-Silva *et al.*, 2021), which performs annotation using MitoFinder ( Allio *et al.*, 2020). The genome was analysed and BUSCO scores were generated within the BlobToolKit environment ( Challis *et al.*, 2020). Table 3 contains a list of all software tool versions used, where appropriate.

**Table 3.** Software tools used.

Software tool	Version	Source
BlobToolKit	3.4.0	( Challis *et al.*, 2020)
freebayes	1.3.1-17-gaa2ace8	( Garrison & Marth, 2012)
gEVAL	N/A	( Chow *et al.*, 2016)
Hifiasm	0.12	( Cheng *et al.*, 2021)
HiGlass	1.11.6	( Kerpedjiev *et al.*, 2018)
longranger align	2.2.2	https://support.10xgenomics.com/genome-exome/software/pipelines/latest/advanced/other-pipelines
MitoHiFi	1.0	( Uliano-Silva *et al.*, 2021)
PretextView	0.1.x	https://github.com/wtsi-hpag/PretextView
purge_dups	1.2.3	( Guan *et al.*, 2020)
SALSA2	2.2	( Ghurye *et al.*, 2019)

### Genome annotation

The icAdaBipu1 genome was annotated using the Ensembl rapid annotation pipeline ( Aken *et al.*, 2016) ( Table 1; https://rapid.ensembl.org/Adalia_bipunctata_GCA_910592335.1/Info/Index). The resulting annotation includes 26,646 transcribed mRNAs from 13,611 protein coding and 3,277 non-coding genes.

### Ethics/compliance issues

The materials that have contributed to this genome note have been supplied by a Darwin Tree of Life Partner. The submission of materials by a Darwin Tree of Life Partner is subject to the Darwin Tree of Life Project Sampling Code of Practice. By agreeing with and signing up to the Sampling Code of Practice, the Darwin Tree of Life Partner agrees they will meet the legal and ethical requirements and standards set out within this document in respect of all samples acquired for, and supplied to, the Darwin Tree of Life Project. Each transfer of samples is further undertaken according to a Research Collaboration Agreement or Material Transfer Agreement entered into by the Darwin Tree of Life Partner, Genome Research Limited (operating as the Wellcome Sanger Institute), and in some circumstances other Darwin Tree of Life collaborators.

## Data Availability

European Nucleotide Archive: Adalia bipunctata (2-spot ladybird), Accession number PRJEB45127, https://identifiers.org/ena.embl:PRJEB45127 ( Wellcome Sanger Institute, 2022). The genome sequence is released openly for reuse. The *A. bipunctata* genome sequencing initiative is part of the Darwin Tree of Life (DToL) project. All raw sequence data and the assembly have been deposited in INSDC databases. Raw data and assembly accession identifiers are reported in Table 1.

## Author information

Members of the Wellcome Sanger Institute Tree of Life programme are listed here: https://doi.org/10.5281/zenodo.4783585.

Members of Wellcome Sanger Institute Scientific Operations: DNA Pipelines collective are listed here: https://doi.org/10.5281/zenodo.4790455.

Members of the Tree of Life Core Informatics collective are listed here: https://doi.org/10.5281/zenodo.5013541.

Members of the Darwin Tree of Life Consortium are listed here: https://doi.org/10.5281/zenodo.4783558.
