# Supplementary material for: The genome sequence of the two-spot ladybird, Adalia bipunctata (Linnaeus, 1758)
Source: Wellcome Open Res. 2022 Nov 30;7:288. [Version 1] doi: 10.12688/wellcomeopenres.18610.1 (PMC10521051; doi:10.12688/wellcomeopenres.18610.1)
Supplement: Peer review history

# Reviewer report: Reviewer response for version 1

**DOI:** 10.21956/wellcomeopenres.20637.r65980

**Published:** 2023-09-27

**Authors:** Shota Nomura

**Affiliations:** 1 Division of Evolutionary Developmental Biology, National Institute for Basic Biology, Okazaki, Japan

This study performed the chromosome-level genome assembly of the two-spot ladybird, *Adalia bipunctata*. Authors assembled the contigs using PacBio single-molecule HiFi long reads and scaffolded them using Hi-C data. As a result, the authors obtained 118 scaffolds with 45.9 Mb of N50 and 97.6% of BUSCO score. Among the assembly, 94.87% were assigned to 9 autosomes and X and Y sex chromosomes, which was consistent with the number of chromosomes observed in *A. bipunctata*.

 Authors found high quality chromosome-level assembly, and the methods of analyses were appropriate and well explained. I consider this manuscript is worthy for publication. I note below a few minor questions.

 1. How many ladybird species have chromosome-level genome sequences been published? I know the harlequin ladybird has been well studied and that genome assembly has been published, but has chromosome-level assembly been done for other ladybird species? Also, does the genome compositions or number of chromosomes in two-spot ladybird are different from other ladybirds that have chromosome-level genome assembly? This information would be useful to readers who use the assembled genome sequences and would be better to be explained in the Background section.

 2. How did authors distinguish X and Y sex chromosomes from autosomes? If authors obtained male and female reads, they can be distinguished based on differences in reads mapping between the sexes. Did the authors use such a method? In any case, it would be better to be explained the methods of distinguish them.

Are sufficient details of methods and materials provided to allow replication by others?

Partly

Is the rationale for creating the dataset(s) clearly described?

Partly

Are the datasets clearly presented in a useable and accessible format?

Yes

Are the protocols appropriate and is the work technically sound?

Yes

Reviewer Expertise:

genomic, ecological evolution, RNA-seq, speciation, Insect, genitalia

I confirm that I have read this submission and believe that I have an appropriate level of expertise to confirm that it is of an acceptable scientific standard.

# Reviewer report: Reviewer response for version 1

**DOI:** 10.21956/wellcomeopenres.20637.r65975

**Published:** 2023-09-15

**Authors:** Themistoklis Giannoulis

**Affiliations:** 1 Laboratory of Genetics, Faculty of Animal Science, University of Thessaly, Larissa, Greece

This is a very well presented research, the results are clearly described and the introduction has the proper length. The authors could provide more information about the bioinformatics processes (maybe as a supplementary file), p.ex from the arguments they used in freebayes etc. Also, since there was only 1 specimen, how were the variants called? Is there a reference genome already available?

Are sufficient details of methods and materials provided to allow replication by others?

Yes

Is the rationale for creating the dataset(s) clearly described?

Yes

Are the datasets clearly presented in a useable and accessible format?

Yes

Are the protocols appropriate and is the work technically sound?

Yes

Reviewer Expertise:

Animal Genetics and Genomics

I confirm that I have read this submission and believe that I have an appropriate level of expertise to confirm that it is of an acceptable scientific standard, however I have significant reservations, as outlined above.

# Reviewer report: Reviewer response for version 1

**DOI:** 10.21956/wellcomeopenres.20637.r65976

**Published:** 2023-09-12

**Authors:** Fahad Alqahtani

**Affiliations:** 1 National Center for Agricultural Technology, National Center for Agricultural Technology, King Abdulaziz City for Science and Technology, Riyadh, Saudi Arabia

The authors successfully sequenced the two-spot ladybird, Adalia bipunctata, in their study titled "The genome sequence of the two-spot ladybird, Adalia bipunctata (Linnaeus, 1758)". They employed three sequencing technologies: PacificBiosciences SEQUEL II, 10X Genomics Illumina, and Hi-C Illumina. The average coverage achieved was 48X for Pacific Biosciences single-molecule HiFi long reads and 80X for 10X Genomics.

 To assemble the two-spot ladybird PacBio reads, the authors utilized Hifiasm, followed by polishing the PacBio contigs using the longranger align tool with 10X Genomics reads. Additionally, they performed scaffolding with Hi-C data using SALSA2. Overall, the genome assembly consisted of 118 sequence scaffolds, representing nine chromosomes and the sex chromosomes (X and Y). The total length of these sequences amounted to 475,288,177 base pairs. The quality of the assembly was assessed using BUSCO and yielded high scores.

 However, there are some minor comments that need to be addressed. Firstly, in the method section, it would be beneficial to mention the closely related species used by the MitoHiFi/Mitofinder tool for guiding the annotation of the mitochondrial genome. Secondly, in the Genome Assembly section, the term "Pretext" should be corrected to "PretextView".

Are sufficient details of methods and materials provided to allow replication by others?

Yes

Is the rationale for creating the dataset(s) clearly described?

Yes

Are the datasets clearly presented in a useable and accessible format?

Yes

Are the protocols appropriate and is the work technically sound?

Yes

Reviewer Expertise:

Bioinformatics

I confirm that I have read this submission and believe that I have an appropriate level of expertise to confirm that it is of an acceptable scientific standard, however I have significant reservations, as outlined above.

# Reviewer report: Reviewer response for version 1

**DOI:** 10.21956/wellcomeopenres.20637.r65973

**Published:** 2023-09-12

**Authors:** Hermes E. Escalona

**Affiliations:** 1 Australian National Insect Collection, CSIRO, Canberra, Australia

This is another great addition by DTOL. I am currently performing phylogenomic and comparative genomic research on ladybirds and other beetles and this resource is invaluable. I think that the manuscript is impeccable with some minor comments, see below.

 - Pl notice that *Harmonia axyridis *was introduced for biocontrol of Aphids worldwide. Certainly, it is an invasive species, but it was initially introduced. If possible please clarify in the Background section.

 - The Genome sequence report mention 10X Genomics and Hi-C data, will it be possible to clarify in which order they were used?

 - I found that approx 800 sequences from the cds annotation file have identical residues e.g. ENSABPT00005022599.1 and ENSABPT00005022609.1. Will it be possible to add a note explaining this and may be also in the annotation README release? I found a higher (<3000) number of sequences with identical residues in the ladybirds icCocSept1.1 and icHarAxyr1 generated by DTOL.

 Please add a few more details on the Manual assembly curation so the methods are clear and reproducible. If not all the details are necessary, if possible at least please provide major guidelines.

Are sufficient details of methods and materials provided to allow replication by others?

Yes

Is the rationale for creating the dataset(s) clearly described?

Yes

Are the datasets clearly presented in a useable and accessible format?

Yes

Are the protocols appropriate and is the work technically sound?

Yes

Reviewer Expertise:

Entomology, insect morphology, beetle taxonomy and systematics and molecular evolution in particular phylogenomics and comparative genomics.

I confirm that I have read this submission and believe that I have an appropriate level of expertise to confirm that it is of an acceptable scientific standard.

# Reviewer report: Reviewer response for version 1

**DOI:** 10.21956/wellcomeopenres.20637.r56737

**Published:** 2023-05-26

**Authors:** Gregor Bucher

**Affiliations:** 1 Johann-Friedrich-Blumenbach-Institute for Zoology and Anthropology, Dept. of Evolutionary Developmental Genetics, Georg-August-Universitat Gottingen, Göttingen, Lower Saxony, Germany

The Tree of Life Programme team presents its efforts to sequence the genome of the two-spot ladybird *Adalia bipunctata*. This species is an important predator of aphids and is currently challenged by invasive ladybirds such that research on this animal is of importance.

 PacBio long reads and 10X genomics read clouds were combined with Hi-C data to sequence and assemble the genome. Based on the high coverage and the combination of techniques, the result is an excellent genome sequence with chromosome level assembly including both sex chromosomes and a BUSCO score of 97,6%. A number of analyses such as GC content were done to characterize the genome further. The annotation revealed a small protein coding gene set (appr. 13.600 genes), which seems very surprising. In summary, a very valuable high quality genome sequence of an insect of importance is provided to the community and will surely be used in many future studies.

 While this clearly is a valuable resource, I think that the paper could do more to inform the reader beyond the fact that an excellent sequence was provided. First, in its current form, the paper simply describes the procedure and results of the sequencing and some rather superficial analyses. I would have appreciated some more effort interpreting and putting the results into context. Some non-comprehensive suggestions: Does the number of scaffolded chromosomes coincide with the cytological data? How does the genome size and GC content relate to other insects and beetles? Any property of the genome that deviates from expectations? Similarly, the results of the analyses shown in the figures are not well explained and not interpreted at all leaving it to the reader to make sense of those findings (see comments below).

 My second concern is the annotation: A well annotated gene set is a very important aspect to make the resource useful for others. In that respect, I remain a bit unsure with respect to the comparably low number of genes. If true, this would represent a surprising case of gene loss, which should be highlighted. However, it could as well be an erroneous number due to a annotation issues. Actually, it appears that not much emphasis has been put into that topic: The annotation procedure is described rather briefly, information on quality control or the optimization of annotation parameters are not given and it appears that no transcriptomcis data nor comparative annotation across species have been used to enhance the annotation. In summary, some more efforts to extract a good gene set would make the resource even more valuable unless such efforts are underway by groups dedicated to that challenge.  

 Finally, the presentation and description of some data could be improved in order to help understanding the analyses.

 The following aspects have remained unclear to me or were hard to read in the current form:

- I remained a bit unsure, how the chromosomes were assembled from the contigs - I guess this was based on Hi-C data?
- **Figure 2** was hard to read for me. It may be more obvious for genomics aficionados - to help the "users" one could make the figure more intuitive. Some suggestions:
  - mark the extend of individual scaffolds with lines (similar to the red line marking the largest scaffold) and number them in order to relate them to the other data
                 - mark the X and Y chromosomes
                 - the use of colors for indicating the length of scaffolds was difficult to interpret and it leads to some weird depictions: The scaffold covering the N50 line seems to belong at the same time to N50 (orange) and larger scaffolds (grey). Does that scaffold really exactly represent the N50 value? Same with N90. Suggestion for an alternative: Mark N50/N90 simply with dotted circles instead of colors and the length of the scaffolds with a line instead of a color.
              - visualized on that scale the GC content seems to be essentially constant – hence, there is not much information that can be gained from the blue circles outside. Does the GC content really not vary along the chromosomes (e.g. in centromeric regions)? I would have loved to know, which chromosomes have the higher AT content (the smaller chromosomes? sex chromosomes?)
- **Figure 3**: I have remained unsure what actually was plotted here. The 118 scaffolds? I did not understand how you map a scaffold to a phylum? Based on homology of the contained protein coding genes? Isn´t it trivial that all scaffolds map to arthropods given that beetles belong to that clade? What do the axes represent? (what is gc coverage – do you mean gc content? what is the axis “ERR6842....”?). An interesting aspect appeared to be that there are scaffolds with clearly lower GC content - but what does that mean? Do these represent the centromers? Or the sex chromosomes? I have to confess that I have remained unsure what I can actually learn from that figure. Either the results are better described and the results interpreted or instead of that plot the GC content is plotted along each chromosome, which might be more informative.
- **Figure 4**: same issue: what is plotted, how do you map scaffolds to phyla, what do I learn?
- **Figure 5**: Please explain better, what is shown here and mark the chromosome scaffolds and name them (same numbering as in Fig. 2), mark the sex chromosomes. Any curious patterns worth mentioning?

Are sufficient details of methods and materials provided to allow replication by others?

Yes

Is the rationale for creating the dataset(s) clearly described?

Yes

Are the datasets clearly presented in a useable and accessible format?

Partly

Are the protocols appropriate and is the work technically sound?

Yes

Reviewer Expertise:

Evolution, gene function, development, functional genomics

I confirm that I have read this submission and believe that I have an appropriate level of expertise to confirm that it is of an acceptable scientific standard, however I have significant reservations, as outlined above.
